# Trivalent Rare Earth Adsorption at Phosphonic Acid Monolayers

**DOI:** 10.1002/cphc.202500429

**Published:** 2025-09-01

**Authors:** Srikanth Nayak, Ahmet Uysal

**Affiliations:** ^1^ Chemical Sciences and Engineering Division Argonne National Laboratory 9700 S Cass Ave. Lemont IL 60439 USA

**Keywords:** adsorptions, interfaces, ions, monolayers, nonlinear optics

## Abstract

The increasing need for rare earth separations requires a detailed understanding of trivalent ion behavior at charged aqueous interfaces. Here, neodymium (Nd) adsorption on Langmuir monolayers of octadecylphosphonic acid (ODPA), a single‐chain phosphonic acid capable of double deprotonation, at the air/water interface, is investigated. Combining sum frequency generation (SFG) spectroscopy with X‐ray fluorescence near total reflection (XFNTR), both the interfacial water ordering and ion density are examined. Under ambient conditions, Nd ions induce enhanced deprotonation of ODPA headgroups, leading to interfacial ion densities as high as 1 Nd per 30 Å^2^. This adsorption behavior arises from a complex interplay between direct electrostatic interactions, ion pairing, and hydration effects, which cannot be fully captured by classical Gouy–Chapman–Stern models. These insights into trivalent ion adsorption mechanisms provide a pathway toward more effective separation processes for rare earth metals.

## Graphical abstract

This study explores neodymium (Nd) adsorption on Langmuir monolayers of octadecylphosphonic acid (ODPA) at the air/water interface. Using sum frequency generation spectroscopy and X‐ray fluorescence near total reflection, how Nd ions enhance ODPA deprotonation and drive interfacial water ordering is revealed. The findings challenge classical models and offer insights into optimizing rare earth separation processes.

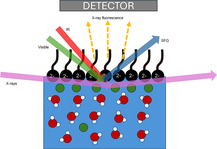

## 1 Introduction

The need for rare earth metals is increasing and will continue to increase for the next decade.^[^ ^1^ ^]^ These seventeen elements (lanthanides plus yttrium and scandium) can be found in earth's crust abundantly, but the difficulty is their separations.^[^ ^2^ ^]^ Most of the rare earths exhibit a trivalent oxidation state and very similar chemical properties leading to their mixed deposit in minerals and making their separations highly challenging. Existing separation technologies try to exploit subtle differences between the rare earth ions, such as lanthanide contraction, to separate them from each other.^[^ ^3^ ^]^ However, the fundamental physics and chemistry of these ions in process conditions are still poorly understood. Developing a better understanding of trivalent ion behavior in aqueous interfaces can provide new insights and directions for better rare earth separations.^[^ ^4^, ^5^, ^6^, ^7^ ^]^

Gouy–Chapman–Stern model is one of the mostly used approaches to describe the distribution of ions near a charged surface.^[^ ^8^, ^9^ ^]^ Unfortunately, this model is limited to monovalent, point‐like charges at low concentrations and ignores solvent effects, such as hydration enthalpy and interfacial water organization.^[^ ^10^ ^]^ Although there have been numerous updates and improvements to the model, we still lack a broader version that describes trivalent ion behavior at charged interfaces in a predictive way.^[^ ^8^, ^11^ ^]^ One difficulty in achieving this goal is the limited direct experimental measurements of trivalent ions at charged interfaces. In addition to the general difficulties of interface specific experiments, rare earths did not get much attention until recently. Therefore, there are many open questions.

One important question is what is the maximum number of trivalent ions that can be adsorbed at a charged interface, an important parameter for the capacity of separation systems. Although in classical mean field theories, the counter ions can only compensate for the surface charge, there have been many experimental observations and theoretical calculations that demonstrated that in certain cases, especially with multivalent ions, it is possible to adsorb more counterions than needed to compensate for the surface charge that might lead to a charge reversal.^[^ ^12^, ^13^, ^14^, ^15^, ^16^, ^17^, ^18^, ^19^, ^20^, ^21^, ^22^, ^23^ ^]^ Interestingly, the definitions of overcharging and/or charge reversal vary in literature based on the method it is detected with and on the context it is discussed. Sometimes, the number of ions detected without knowing the exact charge on the adsorbing species, and sometimes surface charge is probed without directly detecting which ions adsorb. Nevertheless, it is mainly considered that the origin of overcharging can be physical or chemical.^[^ ^12^, ^14^, ^20^ ^]^ The former implies that the charge inversion is mainly driven by the ion–ion correlations and can be described by direct electrostatic interactions. Although it is not always explicitly explained, we suggest that this would mean that the direct electrostatic interactions are significantly stronger to determine the overall behavior. The surface charge density is also an important parameter. The chemical origin typically refers to ion‐pair formation, complexation, or covalent bonding being dominant.^[^ ^14^ ^]^ Lanthanide ions remain trivalent under acidic conditions and some of their behavior can be explained by direct electrostatic interactions. In contrast, other trivalent ions, such as iron, form Fe(OH)^2+^ and Fe(OH)_3_ species even in acidic conditions and their interfacial behavior is dominated by specific and/or covalent interactions.^[^ ^15^ ^]^

**Figure 1:**
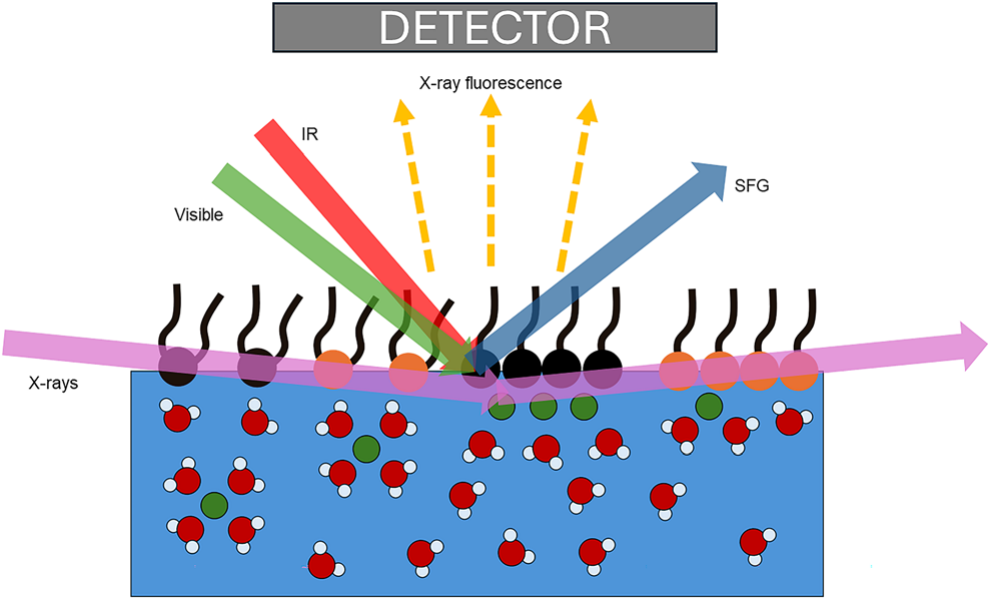
A cartoon depiction of ion adsorption studies at Langmuir monolayers at the air/water interface. Surfactant properties can be controlled by changing the number of alkyl tails and the headgroup properties (depicted as orange or black). Single‐ and double‐chain surfactants typically have 20 and 40 Å^2^ molecular area, respectively. This interface can be accessed by SFG spectroscopy and surface sensitive X‐ray fluorescence techniques.

Langmuir monolayers of amphiphilic surfactants are commonly used to study adsorption at soft charged interfaces.^[^ ^7^, ^11^, ^13^, ^15^, ^19^, ^24^, ^25^, ^26^, ^27^, ^28^ ^]^ They can provide high charge densities and a broad range of tunability in surface functional groups. They can also be accessed with surface specific probes, such as sum frequency generation (SFG) spectroscopy and surface X‐ray scattering and fluorescence techniques (**Figure** **1**).

In SFG spectroscopy, surface charge is inferred from the orientational ordering of the interfacial water molecules. As the counterions adsorb, they compensate for the surface charge and the SFG signal decreases.^[^ ^29^, ^30^, ^31^ ^]^ These signals can be obscured due to additional factors that may affect the interfacial water ordering, such as the specific interactions with the surface or the asymmetric hydration shell of the adsorbed ions, due to partial dehydration at the interface.^[^ ^18^, ^32^ ^]^ Surface specific X‐ray fluorescence near total reflection (XFNTR) can detect the number of ions in the interfacial region directly by taking advantage of their characteristic X‐ray emissions.^[^ ^33^, ^34^, ^35^, ^36^ ^]^ However, this technique is insensitive to the charged state of the ions, leading to ambiguity of the total surface charge. For instance, if hydroxy‐ or chloro‐ complexes of a trivalent ion adsorb at the interface, they might have less than a 3+ charge. Using these two techniques together helps to avoid these ambiguities.

Phosphorus‐based acidic extractants are widely used in rare earth separations.^[^ ^37^, ^38^ ^]^ Model systems of trivalent ion adsorption to phosphate‐based surfactants provide insights to these processes.^[^ ^25^, ^26^, ^27^, ^28^, ^33^, ^39^, ^40^ ^]^ Dihexadecyl phosphate (DHDP) has a phosphoric acid headgroup and two alkyl tails. DHDP can only deprotonate once making it 1‐charged.^[^ ^39^ ^]^ Since it has two tails, molecular area of DHDP films is ≈40 Å^2^.^[^ ^26^ ^]^ It has been shown that light lanthanides, such as La and Nd adsorb to DHDP at 1:3 ratio, compensating the surface charge.^[^ ^15^, ^27^, ^40^ ^]^ Another surfactant that was widely studied is dimyristoylphosphatidic acid (DMPA). The phosphatidic acid headgroup can deprotonate twice making it to have a nominal 2‐charge.^[^ ^13^, ^41^ ^]^ DMPA also has two tails with a molecular area of ≈40 Å^2^. Interestingly, X‐ray studies showed that La ions bind at 1:1 ratio to DMPA.^[^ ^13^ ^]^ This leads to each headgroup‐lanthanum complex to have 1+ overall charge if the ions keep their trivalent state. Although the original X‐ray studies could not determine this directly, independent heterodyne‐detected SFG (HD‐SFG) studies showed that, indeed, the Im *χ* ^(2)^ of the water signal changes from positive to negative, consistent with the charge reversal at the interface.^[^ ^41^ ^]^ The origin of this overcharging was mainly explained as La ions forming contact ion pairs with the headgroup, and not due to the ion–ion correlations. Indeed, carboxylic acid headgroups, with a similar surface charge density do not show any overcharging with light lanthanides,^[^ ^15^, ^18^ ^]^ indicating that the surface chemistry is the main driver in the case with DMPA.

Second deprotonation of DMPA headgroup leads to an interesting question about the highest possible surface charge density of a Langmuir monolayer. Although DMPA headgroups have a 2‐charge, since they have two chains, their surface charge density is similar to that of a single‐chained, single‐deprotonated surfactant (–0.8 C m^−2^). In this study, we investigate Nd adsorption to a single‐chain phosphonic acid monolayer, octadecylphosphonic acid (ODPA), which can also have two deprotonations. If Nd ions form ion pairs with the headgroup in a similar way that they had with DMPA, the Nd ion density can reach as high as 1 ion per 20 Å^2^. Interestingly, Nd adsorption at ODPA monolayers had been studied earlier and Nd ion density reached only up to 1 ion per 60 Å^2^ suggesting only a single deprotonation and simple charge compensation.^[^ ^28^ ^]^ However, that study was conducted at 8 °C. Here, we show that at room temperature Nd ions can deprotonate ODPA headgroups to 2‐ and can adsorb at higher charge densities, but not as high as 1:1 ratio observed with DMPA. We investigate the interfacial ion density using XFNTR and interfacial charge density with SFG spectroscopy.

## 2 Results and Discussion

**Figure 2:**
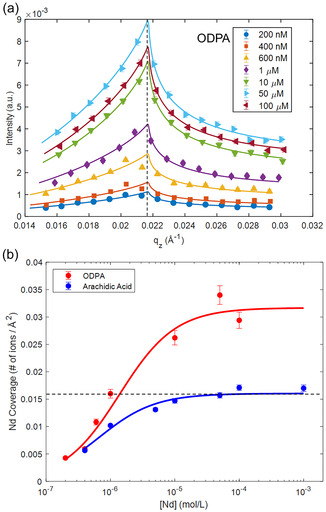
a) XFNTR spectra (symbols) collected at the air/water interface with ODPA surfactant and increasing Nd concentration in the subphase. The lines represent model‐dependent fits explained in the text and the supporting information. The XFNTR signal increases with q_z_ below the critical angle (q_c_, vertical dashed lines), due to the excess interfacial Nd. b) Interfacial Nd coverage calculated from the XFNTR fits plotted as a function of the bulk Nd concentration (red symbols). The data for arachidic acid (blue) is taken from ref. [18] for comparison. The horizontal dashed line corresponds to 63 Å^2^ area per Nd. If all the adsorbed Nd ions are trivalent, it compensates the total charge of −1 charged, single‐chain surfactant with 21 Å^2^ molecular area.

**Figure** **2**a shows the X‐ray fluorescence intensity as a function of the vertical momentum transfer (q_z_) for increasing Nd subphase concentrations. The refractive index of water is less than 1 for water (and most materials).^[^ ^34^ ^]^ Therefore, at very shallow incidence angles less than the critical angle (q_c_), total external reflection occurs (Figure 1). Only the evanescent waves penetrate to water up to 5 nm (for 10 keV X‐ray energy).^[^ ^34^, ^35^ ^]^ Hence, the XFNTR signal below the critical angle only comes from the interfacial ions. Since the Fresnel transmission is proportional with q_z_ under these conditions, the XFNTR signal increases with q_z_ below the critical angle. When q_z_ > q_c_, majority of X‐rays penetrate water. Since the bulk Nd concentration is very low, the XFNTR signal decreases with increasing q_z_, leading to the curves observed in Figure 2a. These data can be modeled to calculate the interfacial Nd density (Experimental Section).^[^ ^33^, ^35^ ^]^ Figure 2b (red trace) shows the calculated interfacial Nd densities as a function of the bulk Nd concentration.

At 200 nm bulk Nd concentration, there are 0.0043 Nd ions per Å^2^ at the interface (Figure 2a, red trace). This corresponds to 1 Nd ion per 232 Å^2^, or per 11 ODPA molecules. The interfacial coverage increases with bulk Nd rapidly and reaches to ≈1 Nd per 3 ODPA molecules at 1 μM bulk concentration. This corresponds to a total charge compensation, if we assume that ODPA molecules are single charged, and all the interfacial Nd ions are trivalent (horizontal dashed lines). Interestingly, as the bulk Nd concentration is increased more Nd ions adsorb at the interface leading to an apparent overcharging. At 50 μm bulk concentration, interfacial coverage reaches and plateaus at 2 Nd per 3 ODPA molecules. This is in stark contrast to the adsorption behavior with arachidic acid monolayers that was investigated in an earlier study,^[^ ^18^ ^]^ in which the Nd adsorption saturates at 1 Nd per 3 ODPA molecules (Figure 2a, blue trace). Both arachidic acid and ODPA have a single hydrocarbon tail with slightly different lengths of 20 and 18 carbons respectively. Both of their molecular areas are around 20 Å^2^.^[^ ^26^ ^]^ This clearly demonstrates that the EDL structure is not purely controlled by the ions in the solution and the main difference should be originating from the carboxylic acid vs. phosphonic acid headgroups, which will be discussed below.

We describe the Nd adsorption as apparent overcharging, because XFNTR is not sensitive to the speciation of Nd nor to the deprotonation level of ODPA. The second pK_a_ of the ODPA headgroup is 7.2.^[^ ^13^ ^]^ However, under similar conditions, DMPA molecules were known to have second deprotonation and reach 2‐charge, as the trivalent ions can effectively exchange the second proton.^[^ ^41^ ^]^ If that is also the case with ODPA, then 2:3 Nd:ODPA ratio corresponds to a charge compensation. To investigate this further, we conducted SFG spectroscopy studies.

**Figure 3:**
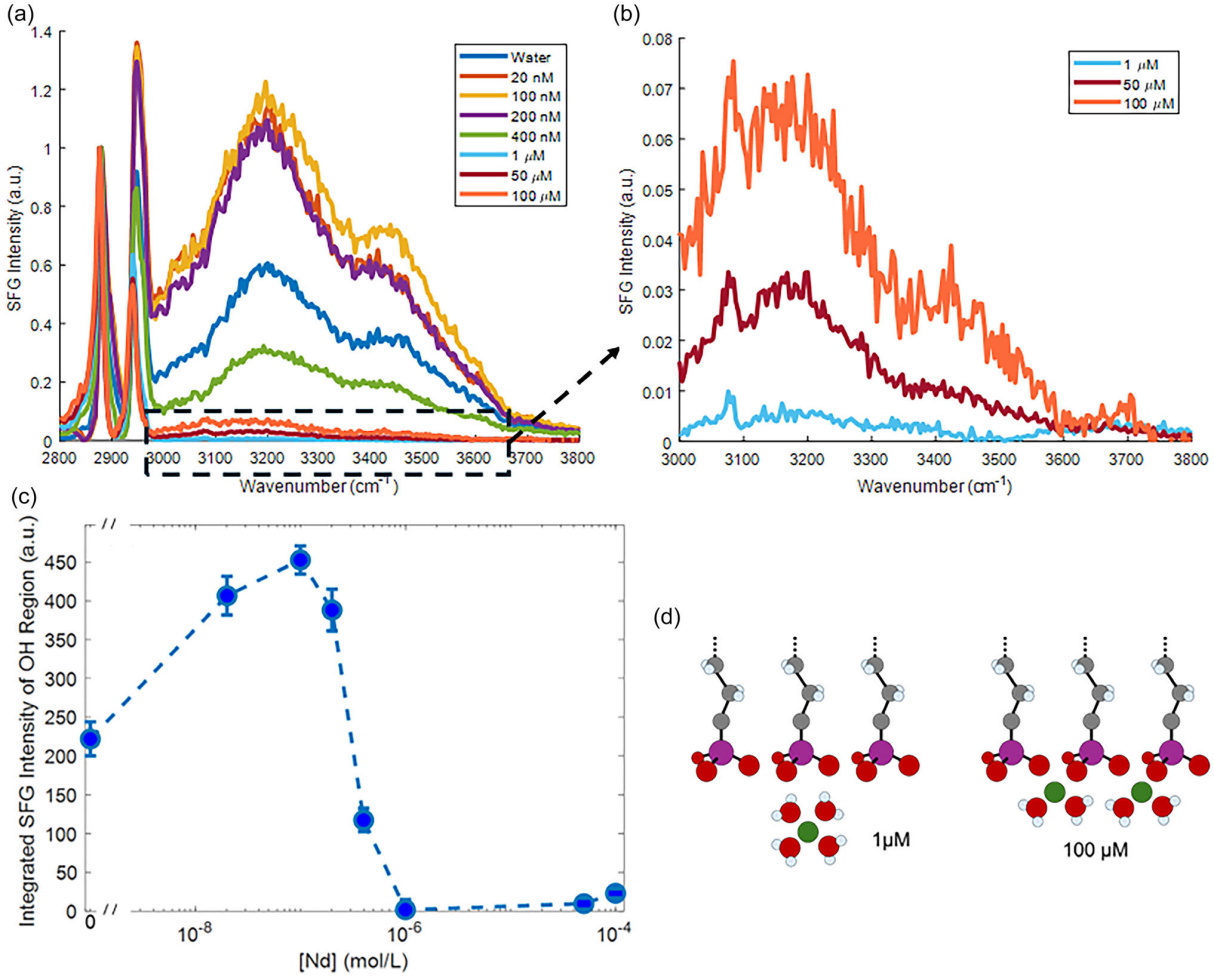
a) SFG spectra collected at the air/water interface with ODPA surfactant and increasing Nd concentration in the subphase. The sharp peaks at 2876 and 2940 cm^−1^ are assigned to the —CH_3_ terminal group and its Fermi resonance, respectively. The Fermi resonance peak appears to blue shift and enhance due to the constructive interference with the —OH signal, for [Nd] < 1 μM. Each spectra intensity is normalized to its 2876 cm^−1^ peak intensity. b) Zoomed in plots of —OH region for [Nd] ≥ 1 μM. c) Integrated SFG intensity of the —OH regions plotted as a function of bulk Nd concentration. Dashed lines are a guide to eye. d) A cartoon depiction of possible interfacial hydration at 1 and 100 μm.

**Figure** **3**a shows the SFG spectra between 2800 and 3800 cm^−1^ for increasing Nd subphase concentrations, with ssp polarization combination (s‐SFG, s‐Visible, p‐IR). The sharp peak at 2876 cm^−1^ originates from the CH_3_ terminal groups of the ODPA tail, and the 2940 cm^−1^ is its Fermi resonance. The absence of CH_2_ signal shows that the ODPA film is well ordered, which leads to a symmetric environment for CH_2_ groups and make them SFG inactive. The absence of a sharp free —OH signal at 3700 cm^−1^ also indicates full coverage of the ODPA film. The 2940 cm^−1^ peak and the broad water signal interferes constructively, since the water molecules are up‐oriented, leading to an increase of this peak for the spectra with stronger —OH signal.

A broad —OH band is observed between 3000 and 3700 cm^−1^, which is mainly dominated by the up‐oriented water molecules due to the negative surface charge created by ODPA. The SFG signal for pure water is weaker than the one for low Nd concentrations (20–200 nM), due to a destructive interference caused by the significantly longer Debye screening length of pure water.^[^ ^19^, ^42^, ^43^, ^44^ ^]^ The decrease of SFG signal above 200 nM is due to the partial screening of the surface charge by the adsorbed ions. The SFG signal drops rapidly above 200 nM and becomes almost 0 around 1 μm bulk concentration. At 50 and 100 μM, SFG signal increases slightly. To see the changes happening in this regime, Figure 3b shows the zoomed in version of these three spectra. Figure 3c shows the integrated SFG intensities plotted as a function of the bulk concentration, to demonstrate this trend more clearly.

The nonmonotonic trend in SFG signals due to the varying Debye length and interference effects had been investigated for monovalent ions in detail.^[^ ^42^, ^43^, ^45^, ^46^, ^47^ ^]^ We have recently employed these ideas to explain monovalent ion interactions with graphene oxide films.^[^ ^44^ ^]^ However, application of this approach to trivalent ions is not straightforward. The validity of Gouy–Chapman model cannot be easily extended to trivalent ions. Also, trivalent ions exchange with protons, complicating the surface charge calculations. Therefore, recent studies with trivalent ions did not model the SFG intensity variations, although they observed similar trends in SFG intensity.^[^ ^19^ ^]^ Nevertheless, we modified and explored these models for trivalent ions (Supporting Information). One major difference between monovalent and trivalent ions is the ionic strength and therefore the Debye length. While the maximum of SFG signal is around 1 mM bulk salt concentrations with monovalent ions,^[^ ^44^, ^47^, ^48^ ^]^ it is around 100 nM with trivalent ions (Figure 3c).^[^ ^19^ ^]^ We can explain this shift by modifying the model developed for monovalent ions (Figure SI2, Supporting Information).^[^ ^47^ ^]^ We note that here we mainly replaced ion concentration with ionic strength but did not attempt to develop a more complex model. The limited dataset does not justify more parameters. Developing a complete model with trivalent ions will require detailed studies, possibly combining SFG with second harmonic generation (SHG) experiments.

The decrease of the SFG signal with increasing concentration and reaching to a minimum around 1 μM corresponds well with the XFNTR data showing that 1:3 Nd:ODPA ratio is reached at this concentration. Therefore, the surface charge is almost zero and the χ^(3)^ contribution becomes very small (Equation (2)). At 50 and 100 μM, SFG signal increases very slightly. According to XFNTR data, Nd coverage is doubled at these concentrations. Therefore, if the ODPA headgroup was singly deprotonated, there should have been significant positive charge, and a very strong SFG signal would be observed due to the χ^(3)^ effect. The absence of such strong SFG signal indicates that indeed Nd ions deprotonate ODPA headgroups and make them effectively 2‐charged. This corroborates well with 2:3 ODPA:Nd ratio and demonstrates that almost all Nd ions are trivalent under these conditions.

The combined interpretation of XFNTR and SFG data provides a complete picture of Nd adsorption at ODPA films that cannot be obtained from a single method. XFNTR provides the total number of ions at the interface but is not sensitive to the charge state of the headgroups. SFG informs about the total surface charge in an indirect way with the overall orientation of the water molecules but cannot detect the metal ions directly. The combined results show that ODPA headgroups are singly deprotonated at low Nd concentrations, and the surface charge leads to a strong SFG signal. When Nd ions compensate the single charge of ODPA with a 1:3 adsorption ratio, the SFG signal almost disappears. As the bulk Nd concentration increases above 1 μm, the SFG signal does not change significantly, but the Nd coverage increases and reaches to a 2:3 ratio with ODPA headgroups. Although this is less than the 1:1 ratio observed with DMPA,^[^ ^13^, ^41^ ^]^ it still leads to 1 Nd per 30 Å^2^ ion density, 33% higher than the 1 La per 40 Å^2^ observed with DMPA. We note that ion per area is not a very robust definition as it ignores the z distribution of the ions. For instance, 1 Y per 11 Å^2^ was observed at electrified graphene interface,^[^ ^16^ ^]^ but in the absence of any functional groups, the ions were distributed over a wide diffuse layer.

These results do not rule out the possibility to observe higher Nd to ODPA ratio under ideal conditions. Unfortunately, single‐chained ODPA is not as stable as DMPA, and we were not able to increase the bulk concentration beyond 100 μM. It might be interesting to study self‐assembled monolayers of similar headgroups fixed to a solid substrate.

The reappearance of SFG signal at 50 and 100 μM concentrations can be further investigated. The sharp feature at 3080 cm^−1^ likely originates from alkene impurities in some of the tail groups of ODPA (Figure SI1, Supporting Information). Interestingly, this feature appears as a dip in water and 400 nm spectra (Figure 3b). It is possible to explain this as the water signal changes sign above 1 μM. Since ODPA is negatively charged, the water molecules orient as hydrogen‐up. Apparently, the SFG signal at 1 μM and above originates from hydrogen‐down water molecules and interfere constructively with the 3080 cm^−1^ peak (Figure 3d). Since the Nd ions directly coordinate to ODPA oxygens, it is very likely that their hydration shell is asymmetric and dominated by hydrogen‐down water molecules. A similar but much stronger increase in SFG signal was observed under carboxylic acid films.^[^ ^18^, ^19^ ^]^ It is likely that the stronger coordination of Nd ions with ODPA headgroups weaken the ordering of the hydration shell waters, leading to a much weaker signal in this case.

## 3 Conclusion

We presented a systematic investigation of Nd ion adsorption to ODPA monolayers at the air/water interface, using XFNTR and SFG spectroscopy. The integrated interpretation of both experimental techniques demonstrated that ODPA surfactants are single deprotonated at low Nd concentrations. When the bulk Nd concentration reaches 1 μM, Nd ions completely compensate the surface charge at 1:3 Nd to ODPA ratio. Further increase in the Nd concentration led to more Nd adsorption that can be explained by second deprotonation of the ODPA headgroups. At 100 μM bulk concentration, we detect 2:3 Nd to ODPA ratio supporting the second deprotonation hypothesis. This leads to 1 Nd 30 Å^2^ surface density, the highest trivalent ion density measured at a Langmuir monolayer.

## 4 Experimental Section

4.1

4.1.1

##### Materials

*n*‐Octadecylphosphonic acid, ODPA, (97%), neodymium(III) chloride hexahydrate (99.9%), hexane (97%), and ethanol (99.5%) are purchased from Millipore Sigma. The salt solutions are prepared by using a Milli‐Q water with 18.2 MΩ resistance. The pH of the 1 mm stock solution was 5.8. The ODPA monolayer was prepared in 9:1 hexane to ethanol mixture as 0.2 mM.

##### XFNTR

Synchrotron experiments were conducted at Sector 15, NSF's ChemMatCARS of Advanced Photon Source at Argonne National Laboratory. XFNTR experiments and methods had been described in detail earlier.^[^ ^33^, ^35^, ^39^ ^]^ Briefly, The X‐ray energy was fixed at 10 keV, which is above the L_3_ absorption edge of Nd (6.208 keV). The X‐ray fluorescence intensity (Nd L_α_ emission at 5.228 keV) is recorded as a function of the vertical momentum transfer, $\left|\vec{q_{z}}\right|$, which is a function of the incidence angle. The energy dispersive X‐ray detector, Vortex‐60EX multi cathode, was placed 10 mm above the surface. The volume of the liquid illuminated by the X‐rays is calculated from the beam dimensions. The footprint of the beam on the liquid surface was always larger than the detector, making only the depth of the illuminated volume a function of the incidence angle. XFNTR data was fit using the XmodFit software.^[^ ^49^ ^]^

**Figure 4:**
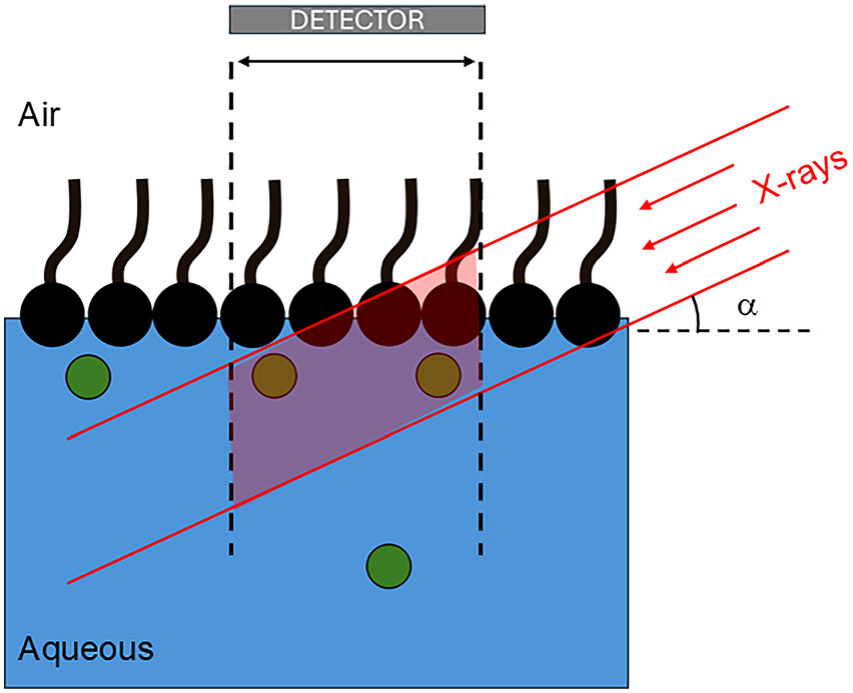
A schematic of XRFNTR measurements at the air/water interface. The shaded region shows the illuminated volume that can be detected by the detector.

The XFNTR method has been explained in detail in the literature.^[^ ^35^, ^39^, ^50^ ^]^ Here, we provide a brief description for completeness. **Figure** **4** shows the experimental diagram. The measured X‐ray fluorescence intensity (*I*_f_) is proportional to the number of ions in the illuminated volume and the incident X‐ray intensity.

(1) If=CI0∫n(z)T(α)e−zΛdV

where *I*_0_ is the incident X‐ray intensity, *C* is a constant of the experimental geometry, *n*(*z*) is the density profile of the neodymium ions, *α* is the incidence angle, *V* is the scattering volume, and Λ is the X‐ray penetration depth.

##### SFG Spectroscopy

Experiments were carried out using a picosecond EKSPLA laser system having a pulse duration of 29 ps and a repetition rate of 50 Hz. Details of the system have been published previously.^[^ ^32^ ^]^ Briefly, a 1064 nm beam from a mode‐locked Nd:YAG laser is split and frequency is doubled to 532 nm inside a harmonic unit. One of the 532 nm beams is directly used to probe the sample while the remaining beams are parametrically combined to generate a tunable IR beam. The beams are overlapped in space and time in a reflection geometry; the visible and IR excitation angles, with respect to the sample normal, are *θ*_vis_ = 60° and *θ*_IR_ = 55°, respectively. The 532 nm polarization is adjusted with a *λ*/2 waveplate, and the SFG signal is selected using a Glan polarizer. After the sample, the SFG signal is directed to a monochromator and collected with a photomultiplier tube (Hamamatsu, R7899). A motorized piezoelectric rotation stage (ThorLabs, ELL8K) rotates the sample after ten frequency steps to avoid sample damage. Each spectrum was collected with a 4 cm^−1^ increment over the range 2800−3800 cm^−1^ and average of 50 laser shots per point. The spectra were normalized against the SFG spectrum of z‐cut quartz. VSFG data were collected with the SSP polarization conditions, where S‐SFG signal, S‐VIS, and P‐IR. ODPA monolayer was prepared in a circular polytetrafluoroethylene (PTFE) dish having a diameter of 6 cm for the SFG experiments. A 0.2 mm ODPA solution was added dropwise using a 1 μL syringe (Hamilton, USA) in a dish containing 10 mL of subphase. Surface pressure of the monolayer was measured using a NIMA pressure sensor. All experiments were performed at a surface pressure of 4 ± 1 mN m^−1^ at room temperature. The surface pressure drops 1–2 mN m^−1^ during the data collection. Back‐to‐back measurements show no significant change in the SFG spectra.

Theory of SFG spectroscopy had been explained in detail in the literature.^[^ ^51^, ^52^, ^53^ ^]^ Briefly, the measured SFG intensity is proportional to the square of the effective second order nonlinear susceptibility ${\left|\chi_{\text{eff}}^{\left(2\right)}\right|}^{2}$.

(2) $$|\chi_{\text{eff}}^{\left(2\right)}{|}^{2}\propto |\chi_{\text{NR}}^{\left(2\right)}+\sum_{\upsilon =1}^{n}\chi_{\text{R,}\upsilon }^{\left(2\right)}e^{i\gamma_{\upsilon }}+\frac{\kappa }{\sqrt{\kappa^{2}+{\left(\Delta k_{z}\right)}^{2}}}e^{i\varphi }\chi^{\left(3\right)}\Phi (0){|}^{2}$$

where $\left(\chi_{\text{NR}}^{\left(2\right)}\right)$ and $\left(\chi_{R}^{\left(2\right)}\right)$ are the nonresonant and resonant second order terms. 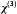 is the third order nonlinear susceptibility, *γ*_ν_ is the $\chi_{R}^{\left(2\right)}$ phase angle, *κ* is the inverse of the Debye screening length, Δ*k*_*z*_ is the inverse of the coherence length of the SFG process, *φ* is the 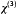 phase angle, and Ф(0) is the surface potential.

## Supporting Information

XFNTR fit parameters, SFG measurements on D_2_O, and a model to explain SFG intensities are reported in the Supporting Information.

## Conflict of Interest

The authors declare no conflict of interest.

## Supporting information

Supplementary Material

## Data Availability

The data that support the findings of this study are available from the corresponding author upon reasonable request.
