# Supplementary material for: Trivalent Rare Earth Adsorption at Phosphonic Acid Monolayers
Source: Chemphyschem. 2025 Sep 1;26(20):e202500429. doi: 10.1002/cphc.202500429 (PMC12530875; doi:10.1002/cphc.202500429)
Supplement: Supplementary file 1 — Supplementary Material [file CPHC-26-e202500429-s001.pdf]

Supporting Information for

Trivalent Rare Earth Adsorption at Phosphonic Acid Monolayers

Srikanth Nayak and Ahmet Uysal*[a]

[a] Dr. S. Nayak, Dr. A. Uysal
Chemical Sciences and Engineering Division
Argonne National Laboratory
9700 S Cass Ave.
Lemont, IL, 60439
E-mail: ahmet@anl.gov

X-ray fluorescence near total reflection (XFNTR) Fit parameters

All XFNTR experiments were conducted at 10 keV X-ray energy. The data was fit by XModFit software.[1] Vertical slit opening was 0.04 mm. y-scale, a unitless normalization constant used for calibration of the detector, obtained by an XFNTR measurement on 20 mM $NdCl_3$ solution without any surfactants and was -6.648844 e-4.

Three parameters were fitted for each XFNTR data set. Q-offset is for the possible offset in the critical angle due to the error in alignment. This is a very small number for all samples and does not affect the final coverage results. Surface coverage is the number of ions per $Å^2$. The ion depth is the distance of the Nd ions from the top of the interface. In this case it is around 20 Å, corresponding to the thickness of the ODPA films.

Bulk Nd Concentration	Surface Coverage (# of ion / $Å^2$)	Ion Depth (Å)	q-offset ($Å^{-1}$)
200 nM	0.0043 ± 0.0002	19.2	-7.02×10^{-4}
400 nM	0.0059 ± 0.0003	19.6	-6.73×10^{-4}
600 nM	0.011 ± 0.0006	17.4	-6.89×10^{-4}
1 µM	0.016 ± 0.001	20.0	-1.25×10^{-4}
10 µM	0.026 ± 0.001	25.9	1.13×10^{-4}
50 µM	0.034 ± 0.001	19.7	3.71×10^{-5}
100 µM	0.029 ± 0.001	22.4	1.95×10^{-4}

Possible origin of the 3080 cm^{-1} peak in SFG spectrum

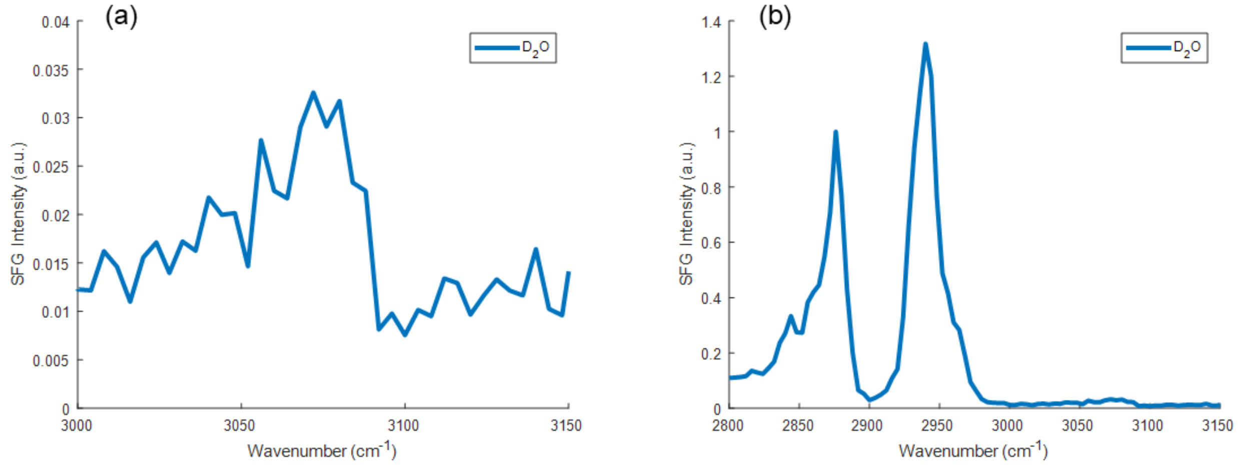

Figure SI-1. (a) SFG spectra collected at the air/D_2O interface with ODPA surfactant shows a very small peak consistent with the similar feature observed in Figure 3 of the main text. (b) The broader range scan to compare the 3080 cm^{-1} peak intensity to CH_3 features.

In Figure 3 of the main text, we suggested that the sharp feature at 3080 cm^{-1} likely originates from ODPA. This suggestion was based on the fact that this feature is not really affected from the solution conditions. However, an ideal ODPA molecule cannot have a vibrational signature at this frequency. All C-H vibraitons of the alkyl tail should be below 3000 cm^{-1}. It is also unlikely that this is a P-O-H vibration as its intensity does not vary with the solution conditions. Especially, it would disappear when most of the headgroups are double deprotonated.

To clarify the possible origins of this peak we spread the ODPA film on D_2O. In the absence of -OH signal we were able to observe a clear 3080 cm^{-1} peak (Figure SI-1), consistent with the intensity observed, either as a dip or as a peak, in Figure 3. This observation strongly suggests that the peak origin is a =C-H vibration, possibly from some surfactants with unsaturated alkene chains. The listed purity of ODPA is 97%. The small amount of impurity is consistent with the very small intensity of 3080 cm^{-1} peak. This impurity in some tail groups does not affect the metal-headgroup interactions.

Modeling Non-monotonic SFG Signal

The non-monotonic trend in SFG signals due to the varying Debye length and interference effects had been investigated for monovalent ions in detail.[2-6] However, application of this approach to trivalent ions is not straightforward. The validity of Gouy-Chapman model cannot be easily extended trivalent ions. Also, trivalent ions exchange with protons, complicating the surface charge calculations. Keeping all of these in mind, here we show that by simply following the model developed for monovalent ions[4] and updating the Debye length calculation, it is possible to capture the main features of the

trend we observe in Figure 3c. We note that this is only an exploration and not intended as a complete description of the system.

Gouy–Chapman theory describes the diffuse electric double layer for a charged surface in contact with an electrolyte solution. The surface charge density σ (C/m^2) is related to the surface potential ψ_0 by the Poisson–Boltzmann equation. For a 1:1 monovalent electrolyte, the Grahame equation gives:

$$\sigma = -\sqrt{8\varepsilon_0\varepsilon_r RTI}\ \sinh\left(\frac{F\psi_0}{2RT}\right),$$

where *I* is ionic strength, F is Faraday's constant, RT is the thermal energy, and ε_r is the dielectric constant of water.

Debye length is defined as

$$\kappa^{-1} = \sqrt{\frac{\varepsilon_0\varepsilon_r RT}{2F^2 I}}$$

Ionic strength is defined as

$$I = \frac{1}{2}\sum_i c_i z_i^2$$

Where c_i is the concentration of ith species and z_i is their valance. Therefore, trivalent ions shrink the diffuse layer more effectively.

The SFG intensity can be written as

$$I_{SF} = I_{vis} I_{IR}\left|\chi^{(2)} + \chi^{(3)}\psi_0\frac{\kappa}{\kappa - i\Delta k_z}\right|$$

Where, I_{vis} and I_{IR} are visible and IR beam intensities, $\chi^{(2)}$ and $\chi^{(3)}$ are second- and third-order susceptibilities, respectively. κ is the inverse of the Debye length above, and Δk_z is a phase factor defined as $\left|k_{viz,z} + k_{IR,z} - k_{SF,z}\right|$. This factor depends on the wavelength and incidence angle of the laser beams. For our system ${\Delta k_z}^{-1} \approx 72$ nm.

The variation in the SFG signal originates from the second term of this equation, due to the variations in the Debye length and the surface potential.

Neglecting the double protonation at below 1 μM Nd concentrations, we can assume three possible surface sites, protonated (θ_H), deprotonated (θ_{free}), and Nd-bound (θ_M). We can write equilibrium relations as follows:

$$A = \frac{K_a}{[H^+]_{bulk}} e^{F\psi_0/RT}$$

$$B = K_M[Nd^{3+}]_{bulk}e^{-3F\psi_0/RT}$$

$$\theta_{free} = \frac{A}{(B+1)(1+A)}$$

$$\theta_M = B \cdot \theta_{free}$$

$$\theta_H = 1 - \theta_{free} - \theta_H$$

Applying the constraint

$$\sigma_{\text{surface}} + \sigma_{\text{diffuse}} = 0,$$

We can numerically solve for the surface potential.

Figure SI2 shows the calculated I_{SF} curve plotted together with the experimental data below 1 μM. The pH was fixed to 5.4 constant, pK_a and pK_M were fixed as 5 and 3 respectively. These numbers are not directly connected to physical parameters, as the model ignores double protonation and Nd replacement with protons. However, the number of data points are very small to justify a more complex model with more parameters. Nevertheless, this model demonstrates that with some reasonable approach it is possible to reproduce the interference effects leading to the maximum at 100 nM bulk concentration.

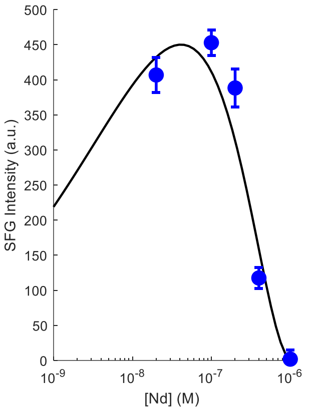

Figure SI-2. Integrated SFG intensity of the -OH regions plotted as a function of bulk Nd concentration, same as the Figure 3c, up to 1 μM bulk concentration (blue symbols). The theoretical I_{SF} curve (solid line), explains the data reasonably.

[1] M. K. Bera, W. Bu, 10.5281/zenodo.7047224, Zenodo, **2022**.

[2] A. P. Fellows, Á. D. Duque, V. Balos, L. Lehmann, R. R. Netz, M. Wolf, M. Thämer, Sum-Frequency Generation Spectroscopy of Aqueous Interfaces: The Role of Depth

and Its Impact on Spectral Interpretation, J. Phys. Chem. C 128 (2024) 20733-20750.
[3] M. D. Boamah, P. E. Ohno, F. M. Geiger, K. B. Eisenthal, Relative permittivity in the electrical double layer from nonlinear optics, J. Chem. Phys. 148 (2018).
[4] E. Tyrode, R. Corkery, Charging of carboxylic acid monolayers with monovalent ions at low ionic strengths: Molecular insight revealed by vibrational sum frequency spectroscopy, J. Phys. Chem. C 122 (2018) 28775-28786.
[5] D. K. Hore, E. Tyrode, Probing Charged Aqueous Interfaces Near Critical Angles: Effect of Varying Coherence Length, J. Phys. Chem. C 123 (2019) 16911-16920.
[6] G. Gonella, C. Lütgebaucks, A. G. F. de Beer, S. Roke, Second Harmonic and Sum-Frequency Generation from Aqueous Interfaces Is Modulated by Interference, J. Phys. Chem. C 120 (2016) 9165-9173.
